# Science in Dentistry.—A Warning

**Published:** 1907-09

**Authors:** Francis Ashley Faught

**Affiliations:** Philadelphia, Pa. Assistant to the Prof. of Clinical Medicine, Medico-Chirurgical College and Hospital.


### SCIENCE IN DENTISTRY.—A WARNING.

By Francis Ashley Faught, M. D., Philadelphia, Pa.

Assistant to the Prof. of Clinical Medicine, Medico-Chirurgical College and Hospital.

The following quotations with comments, taken from a recent article in current dental literature, entitled "Are the Digestive Disturbances of Teething Infants Reflexes or Not?" Will, I think, serve as an illustration of the state of affairs existing in dental literature at the present time, and which was so forcefully commented upon and discussed at the last meeting of the Section of Stomatology of the American Medical Association, at Atlantic City in June.

It is not my purpose to discourage the recent graduate and struggling author from writing and reading papers, that I have seen fit to single out this particular article for criticism, from among the many which are constantly appearing in dental literature throughout the land; but rather to suggest and request that, when one intends to appear in public and permit his statements to appear in print, he will for the sake of those who read to learn and study, if not for his own satisfaction, be sufficiently sure of his facts, that the premises arranged and the conclusions deduced may be logical and convey truth instead of error.

Further, it devolves upon the editorial staff of every journal to carefully read and thoughtfully cull their manuscripts, that their particular organ may be one of usefulness and uplift to all who read, and not accept for any reasons of sentiment any and all material that may reach their desk.

"The theory of reflex nervous action generally held is that the advancing tooth exerts direct pressure on the fibrous gum tissue and that this is sufficient to set up grave constitutional disorders through reflex nervous action and some observers hold that there is also counter pressure on the nerve tissue at the incomplete root apex of the erupting tooth and that this in itself is sufficient to cause trouble."

"If the reflex nervous action theory is true, why is it abolished when the child disrupts the permanent teeth."

The reflex nervous theory is undoubtedly generally held to be AN EXCITING (not "in itself sufficient to cause trouble") cause of many of the digestive disturbances of teething infants. Authority bears out this statement. Gorgas, in "Principles and Practice of Dentistry," page 165, says that, "A perfectly healthy child, properly cared for, may erupt teeth with little or no suffering," and hence little or no reflex disturbances.

In plain words the answer to the Doctor's question is this: Because the infant of six months, one year, or 15 months, is not the infant of 2 or 4, or the child of 6, 12 or 15 years, from either an anatomic, physiologic or pathologic standpoint.

Thomas Morgan Rotch, Professor of Diseases of Children at Harvard, in an article entitled, "Disease and Prevention of Disease in Early Life" (Monthly Cyclopedia of Practical Medicine, May 1907, page 193) states. "It is not only in intrauterine life that development plays such a great role in disease. As soon as the infant is born, even if it is healthy, the question of what changes should take place according to its age immediately arises, and a knowledge of the changes is of greatest importance in the prevention of disease."

"What is normal at birth becomes abnormal if found at one month of age, or again at six months, twelve months or the years. Thus there should be a constant change in the tissues, month after month, and year after year, until the child attains the completed development of the adult. * * * This developmental sequence is found in all tissues and organs of the body and makes the developmental class of disease, and entity, and entirely different from disease as it appears in adult life."

In a healthy infant, properly cared for, the disturbances incident to primary dentition, are very slight, since the eruption of teeth is essentially a physiologic process, hence only when dentition is premature, the child lowered in vitality, or possessing constitutional weakness, or when it is actually suffering from some other disease, do we find the evidence of exaggerated reflexes and the various resulting disturbances which are familiar to all.

From a developmental and anatomic standpoint the following quotations from Gorgas, (loc. cit.) offers a perfectly sound and logical explanation of the existence of the hypersensitivity of the dental (eruptive) reflex.

"Owing to the preponderence of the nervous system in infancy, there is greater sympathy between distant organs than in adult life. * * * The brain is proportionately larger and less perfect in structure."

It is very plain then, that the altered condition of the child due to the natural developmental processes, at the time of eruption of the permanent teeth, renders the organism less susceptible to the disturbances of second dentition.

"In the infant, the digestive, absorbent, and capillary system, are more completely developed than the nervous muscular or osseous, and there's a reason, for there must be a very rapid growth of new tissues besides repair of waste, and consequently the digestive and associated organs, do a comparatively larger amount of work, and that means consequently a large and free flowing blood supply to bear material to the cells."

Apart from the fact that I cannot see the relevency of the wonderful ideas embodied in this paragraph, I fail also to comprehend the meaning of the term "absorbent and capillary system." And further I doubt the truth of the statement that the digestive system is more fully developed than the nervous, muscular or osseous.

"The saprophitic bacteria are essential to life and while innocuous within certain limits, they can become pathologic through excessive numbers."

This short sentence contains two misstatements, in the light of recent experimental research.

First.—Recent experimental clinical studies have shown that bacterial putrefaction and decomposition are essential to neither life or health. Secondly.—Gastrointestinal diseases are not usually caused by an increase in the absolute quantity of bacteria in the intestinal tract (overwhelming the individual), but by either an increase in virulence of existing bacteria, the introduction of not normally present pathogenic bacteria, or through the primary lowering of vital resistance by other disease.

According to J. Duttin Steele (Jou. A. M. A., Aug. 24, 1907, page 647). The total variations in the actual number of bacteria by weight under any circumstances is not more than 15 per cent, usually not more than nine. Surely not an overwhelming increase. He further states that disturbed digestive conditions usually result not from the presence of the bacteria in the tissues, but from the absorption on the soluble toxins formed by their activity.

"To the leukocytes or white blood corpuscles belongs the function among other things restraining and keeping order the putrefactive bacteria."

Nothing could be more absurd. There is no possible way in which the leukocytes could perform the alleged function upon the saprophytic bacteria in the aliamentary canal. Unless there be a distinct break in the continuity of the mucous membrane of the digestive tube, a rare occurence except in special diseases as ameobic or bacillary dysentery, typhoid fever, specific ulceration, etc., which would admit the micro-organisms to the intercellular lymph spaces. Normally the leukocytes do not and cannot reach the contents of the alimentary tract. The leukocytes only perform their function of phagocytosis by surrounding an engulfing the invading bacteria. It is evident then that this "restraining and keeping in order" is a myth without fact or foundation. On the contrary it is a well recognized and established fact that the control of the putrefactive bacteria in the gastro-intestinal tract is dependent in part upon the character of the food and the activity of peristalsis, and that the bacteriacidal agents within this tract are the essential juices of the various digestive glands.

"The digestive organs are in a full blooded state and as the tooth starts to erupt, nature sends a reflex to the digestive organs to get ready for harder work for the teeth are coming, the reflex causes an increase in blood supply to these organs already full. At this time the hygiene of the mouth is usually neglected on account of local tenderness, now should the abdominal surface become chilled we have the cutaneous blood supply driven into the deeper organs and they become congested and the white blood corpuscles are constricted in their movements and a result of this combination of causes the putrefactive bacteria increase in overwhelming numbers (overwhelming increase fallacious) and take charge of the aliamentary canal and nature tries to throw them off through diarrhoeas, etc."

This exceedingly involved paragraph is such a work of art that it is difficult to decide where to begin. The increase in blood supply resulting from the reflex, contrary to the essayists statement, would rather then to augment the circulation of the digestive tract, the effect of which would be to stimulate, not impede, leukocytic activity. The mere matter of increased or decreased leukocytic activity besides would in no way effect the bacterial contents of the digestive tube for the reasons stated above. Further the very fact of the increase of the blood supply would increase the functional activity of the secreting glands of this tract, increasing the flow of bile, etc., which, owing to its antiseptic action, would tend rather to decrease bacterial growth.

The recommendation concerning the wearing of an abdominal band follows the usual teaching, but recently physicians, particularly pediatrists, are coming to the conclusion that proper protection of the extremities is of more importance than all the abdominal bands, since it is the former which usually suffers from changes of temperature, and are far more productive of internal circulatory disturbances than is the abdomen which even without the binder, is adequately covered by the usual infants clothing.

5231 Baltimore Ave.

